# Cold responses and hormonal echoes: a comprehensive view of Raynaud’s vascular dysfunction

**DOI:** 10.1007/s10787-025-01792-0

**Published:** 2025-06-12

**Authors:** Manal Fardoun, Odette El Ghawi, Christie Dib, Leen Jaradi, Marie Therese Chaddad, Hassan Dehaini, Ali H. Eid

**Affiliations:** 1 Faculty of Medicine, American University of Beirut, Beirut, Lebanon; 2 Faculty of Medicine, University of Balamand, El Koura, Lebanon; 3 Case Western Reserve University, The MetroHealth System, Cleveland, OH USA; 4 Department of Basic Medical Sciences, College of Medicine, QU Health, Qatar University, PO Box 2713, Doha, Qatar

**Keywords:** Cold-induced constriction, Arterioles, Peripheral vascular disease, Adrenergic receptors, Thermoregulation

## Abstract

Raynaud’s phenomenon is a peripheral vascular disorder characterized by exaggerated vasoconstrictive response to certain stimuli, most typically cold exposure and emotional stress. Interestingly, Raynaud’s phenomenon incidence is significantly higher in premenopausal females compared to age-matched males, highlighting a role of the female hormone, estrogen, in Raynaud’s phenomenon pathogenesis. Indeed, estrogen plays a fundamental role in potentiating the expression and function of α_2C_ adrenoceptor (α_2C_-AR), the sole mediator of local cooling-induced vasoconstriction. Due to the mosaic nature of Raynaud’s phenomenon involving vascular, hormonal, and neuronal factors, as well as due to the lack of an appropriate animal model, the pathogenesis of Raynaud’s phenomenon is not fully elucidated. Consequently, despite various therapeutic approaches aimed at mitigating symptoms of Raynaud’s phenomenon, a definitive treatment for Raynaud’s phenomenon is quite challenging and remains an unmet need. Therefore, a better understanding of the underlying pathophysiologic mechanisms of Raynaud’s phenomenon is crucial to better delineate pharmacotherapeutic targets to help fight this elusive disease. In this paper, we dissect the molecular and cellular mechanisms underlying Raynaud’s phenomenon and its risk factors, and we shed more light on the role of estrogen. We also explore traditional and current therapeutic approaches, including pharmacologic and non-pharmacologic treatments. In addition, we discuss how the advancement in molecular research offered promising avenues of Raynaud’s phenomenon treatment, namely drug repurposing and molecular targeting. Nonetheless, enhanced awareness, precaution, and good patient compliance are critically important in preventing the progression of Raynaud’s phenomenon and reducing its severity.

## Introduction

Raynaud’s phenomenon is a peripheral vascular disease characterized by episodic vasospastic attacks of peripheral blood vessels, primarily affecting extremities like fingers and toes (Wigley and Flavahan 2016). Other areas, such as nose and nipples, may also be affected (Pauling et al. 2019). A classic manifestation of Raynaud’s phenomenon is a triple color change (pallor, cyanosis, and erythema) attributed to vascular events: initial vasospasm causing white-pallor discoloration, followed by deoxygenation resulting in blue-cyanotic coloration, and finally reperfusion hyperemia resulting in redness (Maverakis et al. 2014; Herrick and Wigley 2020). Severe cases involve puffiness, numbness, or ulceration of digits significantly impacting quality of life (Wigley and Flavahan 2016; Saban et al. 1991). Clinically, one is diagnosed with Raynaud’s phenomenon if they experience at least a biphasic color change (Herrick and Wigley 2020).

Raynaud’s phenomenon affects 3–5% of the general population, reaching up to 10% in some reports (Haque and Hughes 2020; Garner et al. 2015). The epidemiology of this condition varies widely, and is influenced by the mode of definition of Raynaud’s phenomenon, as well as climatic and geographic factors. Expectedly, cold geographic regions are associated with a higher prevalence of Raynaud’s phenomenon (Plissonneau Duquene et al. 2015). Moreover, Raynaud’s phenomenon is a sex-biased condition with a remarkably higher prevalence in premenopausal females as compared to age-matched males (Garner et al. 2015; Fardoun et al. 2016).

Raynaud’s phenomenon is classified into primary or secondary types. Primary Raynaud’s phenomenon is idiopathic, accounts 80–90% of cases, has a better prognosis and lacks severe long-term consequences (Roustit et al. 2014; Haque and Hughes 2020). In contrast, secondary Raynaud’s phenomenon often leads to serious complications such as digital ulcers and ischemia, and is linked to underlying conditions such as cancer and autoimmune diseases, including lupus, rheumatoid arthritis, and scleroderma (Prete et al. 2014). In addition, cases of secondary Raynaud’s phenomenon have been reported in patients taking certain drugs such as beta-blockers and some chemotherapeutic agents (Herrick 2017).

Diagnosis of Raynaud’s phenomenon relies on patient history, clinical assessment, and laboratory tests (Choi and Henkin 2021). Differential diagnosis primarily depends whether Raynaud’s phenomenon is idiopathic or secondary. The patient’s age is another factor contributing to differential diagnosis. While primary Raynaud’s phenomenon affects younger females (aged between 15 and 30 years) who have a positive family history (Temprano 2016; Belch et al. 2017), secondary Raynaud’s phenomenon predominantly affects females who are 40 years of age or older (Haque and Hughes 2020). In men, Raynaud’s phenomenon often occurs later in life due to occupational exposure to vibratory tools or a peripheral vascular condition (Haque and Hughes 2020).

The pathogenesis of Raynaud’s phenomenon is multifaceted, involving vascular, neural, and immunologic components (Wigley 2002). Our incomplete understanding of the pathophysiological processes and the absence of a conclusive treatment for Raynaud’s phenomenon underscore the urgent need for comprehensive research. It is essential to disentangle the intricate mechanisms of Raynaud’s phenomenon to pave the way for targeted and effective treatments that address the specific molecular factors implicated in the disease (Fardoun et al. 2016). This paper reviews current understanding of Raynaud’s phenomenon, focusing on its pathophysiology, risk factors, with a focus on the role of estrogen, while also discussing current and emerging therapeutic approaches and management challenges.

## Pathogenesis of Raynaud’s phenomenon

Cold-induced vasoconstriction at the level of the extremities is a normal physiologic reaction that that aims to redirect blood to internal more vital organs, thus reducing heat loss (Charkoudian 2010). This reflex reaction is mediated by the sympathetic nervous system via the release of norepinephrine (Charkoudian 2010), and via local effectors, namely adrenoceptors at the level of arterioles (Johnson and Kellogg 2010; Chotani et al. 2000). As such, cold-induced vasoconstriction is achieved via the interplay among neuronal, hormonal, and vascular effectors (Wigley 2002). The impaired function of any of these effectors could precipitate an exaggerated vasoconstriction that would manifest as Raynaud’s phenomenon (Herrick 2012; Easter and Marshall 2005). In this section, the vascular element, both at the physiologic and the mechanistic levels, is discussed, and hence, we highlight the roles of arteriovenous anastomosis (AVA) and α_2C_-adrenoceptors in cold-induced vasoconstriction.

### Role of AVA and neuropeptides

Cold-induced vasoconstriction affects localized areas such as fingers, toes, ears, nose tip, and nipples. These areas play a well-orchestrated thermoregulatory function owing to their richness in AVA, which are specialized vascular structures that enable a direct connection between arterioles and venules, bypassing capillaries (Fardoun et al. 2016; Flavahan 2015; Temprano 2016; Lossius et al. 1993). As such, they cannot transport nutrients to the tissues; their function is limited to “transporting” heat to these tissues (Walloe 2016). AVA execute this thermoregulatory function by rapidly adjusting blood flow especially that they are densely innervated by sympathetic adrenergic axons (Walloe 2016; Donadio et al. 2006). As a reflex reaction to cold, AVA act as sphincters, enabling the complete closure of the vessel to preserve body heat (Walloe 2016; Flavahan 2015). In Raynaud’s phenomenon patients, the sympathetic nervous system, which regulates AVA function, becomes hyperactive leading to an exaggerated vasoconstrictive response, reducing blood flow and causing the characteristic triple color change and vasospastic attacks (Temprano 2016; Flavahan 2015). This heightened response may also impair nutritional capillary blood flow, leading to hypoxia and ulcers (Flavahan 2015; Herrick and Wigley 2020).

Initially, the amplified arteriovenous constriction was attributed to functional changes in vascular activity without structural alterations (Herrick and Wigley 2020). However, we now know that secondary Raynaud’s phenomenon is often linked to immune-mediated vascular fibrosis disrupting the balance between vasodilation and vasoconstriction, favoring vasoconstriction (Temprano 2016; Bakst et al. 2008). These vascular abnormalities reduce responsiveness to vasodilatory mediators like nitric oxide and prostacyclin while increasing sensitivity to vasoconstrictors like endothelin-1 (O’Connor 2001). This precipitates a compromised nutritional blood flow and subsequently cutaneous hypoxia. Thus, both structural and functional vascular abnormalities contribute to Raynaud’s phenomenon severity (Herrick and Wigley 2020; Temprano 2016).

Neuronal messengers also play a role in Raynaud’s phenomenon pathophysiology. In response to changes in the microenvironment, neurons release neuropeptides that control vascular tone, maintaining a vasodilation-vasoconstriction balance (Morris 1995). For instance, calcitonin-gene related peptide mediates vasodilation, but its levels are reduced in Raynaud’s phenomenon patients, impairing neural-stimulated vasodilation (Kee et al. 2018; Herrick 2005). Conversely, neuropeptide Y, which induces vasoconstriction in health individuals during mild cooling, is elevated in Raynaud’s phenomenon due to heightened sympathetic activity (Del Carmen Gonzalez-Montelongo et al. 2023; Generini et al. 2005). However, the exact roles of these peptides in Raynaud’s phenomenon remain incompletely understood.

### Role of α_2C_-adrenoceptors

**Fig. 1:**
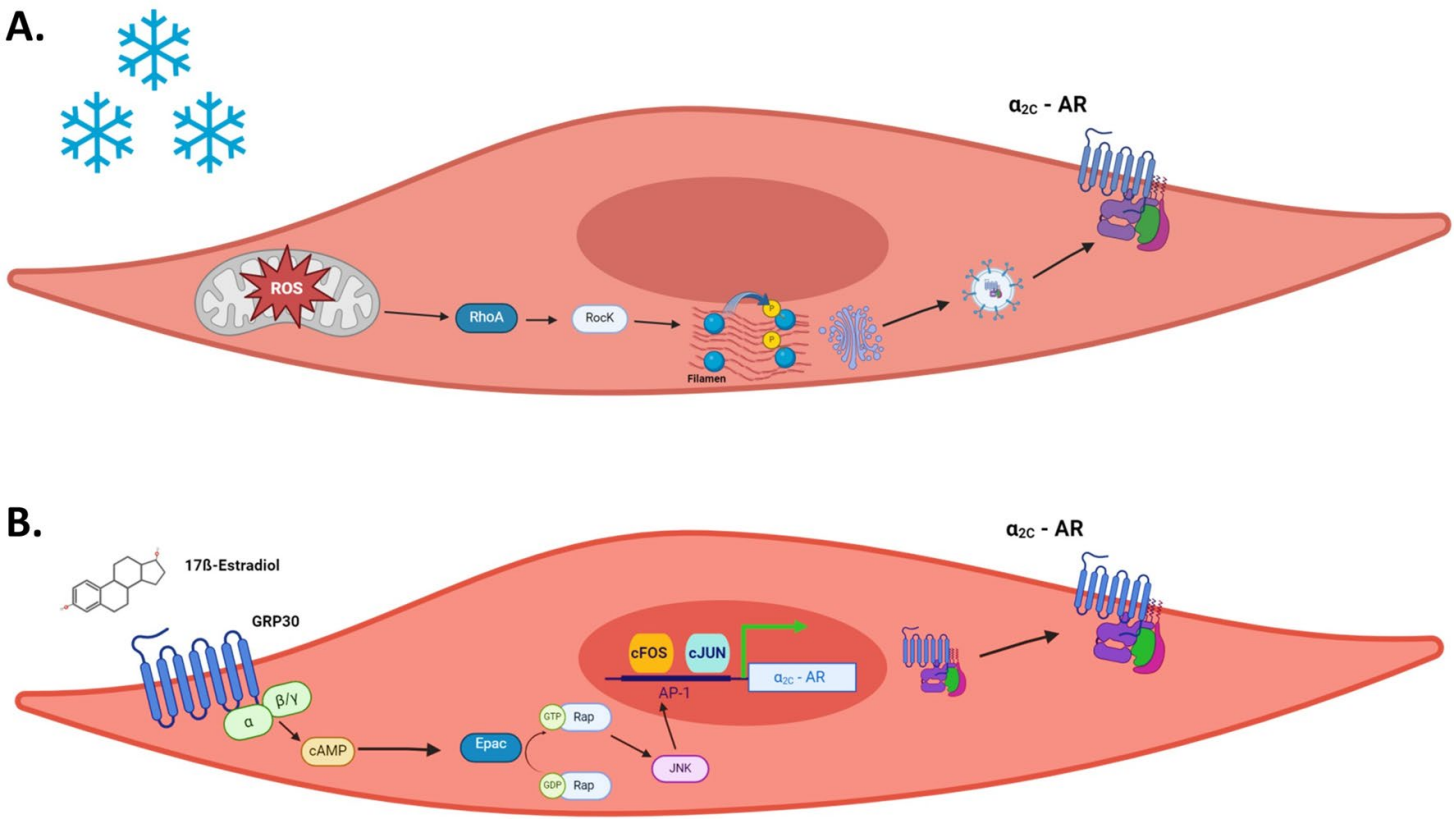
**A** Mechanism of cold-induced mobilization of α_2C_-AR. The mitochondria in arteriolar VSMCs act as thermo-sensors, and release ROS in response to cold. ROS then activates the Rho/ROCK signaling pathway leading to the filamin engagement and actin filament rearrangement. This cytoskeletal architectural change facilitates the trafficking of α_2C_-AR from the Golgi/ER to the cell surface. **B** GPER mediates estrogen-induced α_2C_-AR upregulation through the cAMP/EPAC/JNK/AP-1 pathway. Estrogen, via binding to its membrane receptor, GPER, increases levels of cAMP, which then activates Epac and, in turn, Rap. This active Rap then induces JNK phosphorylation, leading to the dimerization of the c-Fos and c-Jun to form the AP-1 transcription factor, which binds to AP-1 site in the α_2C_-AR promoter and initiates transcription

Cold-induced vasoconstriction is mediated by augmented activity of arteriolar adrenoceptors. Adrenergic receptors on arteriolar smooth muscle cells belong to the α_1_, α_2_ and β_2_ subfamilies (Ahles and Engelhardt 2014). While β_2_-ARs mediate vasodilation, α_1_-ARs and α_2_-ARs are involved in vasoconstriction (Sorriento et al. 2011). Among these, α_2_-ARs are particularly involved in thermoregulation and cold-induced vasoconstriction (Johnson and Kellogg 2010). These receptors are classified into three subtypes: α_2A_, α_2B_, and α_2C_ (MacDonald et al. 1997). α_2A_-AR and α_2B_-ARs do not play a major role in local cooling-induced vasoconstriction, while α_2C_-ARs are the major mediators of this phenomenon (Chotani et al. 2000). Initially, considered vestigial due to their intra-cellular compartmentalization in the Golgi/ endoplasmic reticulum (Golgi/ER), α_2C_-ARs were deemed non-essential, as evidenced by normal viability in α_2C_-AR knockout mice (Sallinen et al. 1997). Interestingly, we and others reported later that α_2C_-AR can be spatially and functionally rescued by physiologic or pathophysiologic stimuli, such as cold or inflammation (Chotani et al. 2000; Bailey et al. 2004; Eid et al. 2008). Cold is sensed by mitochondria in VSMCs; these organelles act as thermosensors, triggering release of reactive oxygen species (ROS), and consequently activating the Rho/Rho kinase signaling pathway (Bailey et al. 2004, 2005; Fardoun et al. 2025). This activation induces the translocation of α_2C_-AR from the Golgi/ER to the plasma membrane (Fig. 1A). There, α_2C_-AR bind adrenaline, initiating cold-induced vasoconstriction.

The spatial rescue of α_2C_-AR in arteriolar smooth muscle cells involves cytoskeletal engagement. Particularly, activation of the Rho/Rho kinase pathway increases F-actin polymerization (Khouri et al. 2016). This F-actin in turns interacts with the actin binding protein, filamin, which helps it bind or rather “carry” α_2C_-ARs, translocating them to the cell surface. This enables α_2C_-ARs to readily bind agonists and precipitate VSMC contraction, leading to vasoconstriction (Pawlowski et al. 2014).

### Raynaud’s phenomenon and estrogen

Overwhelming evidence links estrogen, the female hormone, to Raynaud’s phenomenon (Garner et al. 2015; Fardoun et al. 2024). This conclusion is inferred from several large-scale epidemiologic studies showing a higher incidence of Raynaud’s phenomenon in females compared to age-matched males (Fardoun et al. 2024; Brand et al. 1997; Fraenkel et al. 1999; Harada et al. 1991; Roman Ivorra et al. 2001). For instance, the prevalence of primary Raynaud’s phenomenon ranged from 2 to 20% in women and 1 to 12% in men (Curtiss et al. 2024; Garner et al. 2015; Belch et al. 2017; Maricq et al. 1997). Among women, Raynaud’s phenomenon is more common in premenopausal than post-menopausal individuals (Greenstein et al. 1996). In addition, post-menopausal women on unopposed estrogen-replacement therapy (ERT) are at higher risk than those not using ERT (Fraenkel et al. 1998). These observations incriminate estrogen as a key sex-specific factor in Raynaud’s phenomenon pathogenesis.

We have been studying the signaling mechanisms underpinning cold-induced vasoconstriction, particularly the role of estrogen. We have cemented the conclusion that estrogen upregulates the expression of α_2C_-AR, the sole mediator of local cold-induced vasoconstriction. This indeed explains the higher incidence in females. We have further elucidated the pathways involved, and recently reported that estrogen upregulates α_2C_-AR expression via an EPAC-mediated JNK/AP-1- dependent mechanism (Fardoun et al. 2020) (Fig. 1). This aligns with our previous reports that activation of the cytoplasmic estrogen receptors, ERα and ERβ, upregulates α_2C_-AR in cutaneous VSMCs (Eid et al. 2007). Intriguingly, since estrogen’s effects can be mimicked by the cell-impermeable form of the hormone (estrogen conjugated with bovine serum albumin), we investigated the role of the membrane estrogen receptor, G-protein-coupled estrogen receptor (GPER). Our results show that it is via GPER that estrogen upregulates α_2C_-AR through the cAMP/EPAC/JNK/AP-1 pathway (Fardoun et al. 2023).

Estrogen’s role in modulating vasoreactive responses is well-established in both murine and human vessels. Evidence shows that estrogen potentiates vasoreactivity (Lahm et al. 2008), with estrogen-replete women and female rats exhibiting greater reactivity than their age-matched male counterparts (Li et al. 2014). Notably, estrogen supplementation in male rats augments their vascular responsiveness and premenopausal females show higher reactivity during the mid-menstrual cycle, when estrogen levels peak (Greenstein et al. 1996; Li et al. 2014). Beyond vasoreactivity, estrogen also regulates body temperature (Baker et al. 2020; Zhang et al. 2020). This dual vasculo-thermoregulatory function logically implicates estrogen in Raynaud’s phenomenon, a disorder characterized by aberrant vascular thermoregulatory control.

Collectively, such a preponderance of evidence bespeaks a salient gender-based proclivity in Raynaud’s phenomenon prevalence and clearly show that being a female is tantamount to a Damoclean sword vis-à-vis Raynaud’s phenomenon susceptibility (Garner et al. 2015). Thus, understanding the molecular mechanisms through which estrogen orchestrates Raynaud’s phenomenon onset and pathogenesis is paramount as it will likely contribute to the development of targeted therapies. For instance, drugs that modulate estrogen receptors or impinge upon its signaling cascades may well usher in a new epoch in Raynaud’s phenomenon management, particularly in premenopausal females. Indeed, the role of estrogen and its receptors, particularly GPER, as a potential therapeutic target for Raynaud’s phenomenon has been previously discussed by our group (Fardoun et al. 2023, 2022).

## Raynaud’s phenomenon risk factors

**Fig. 2:**
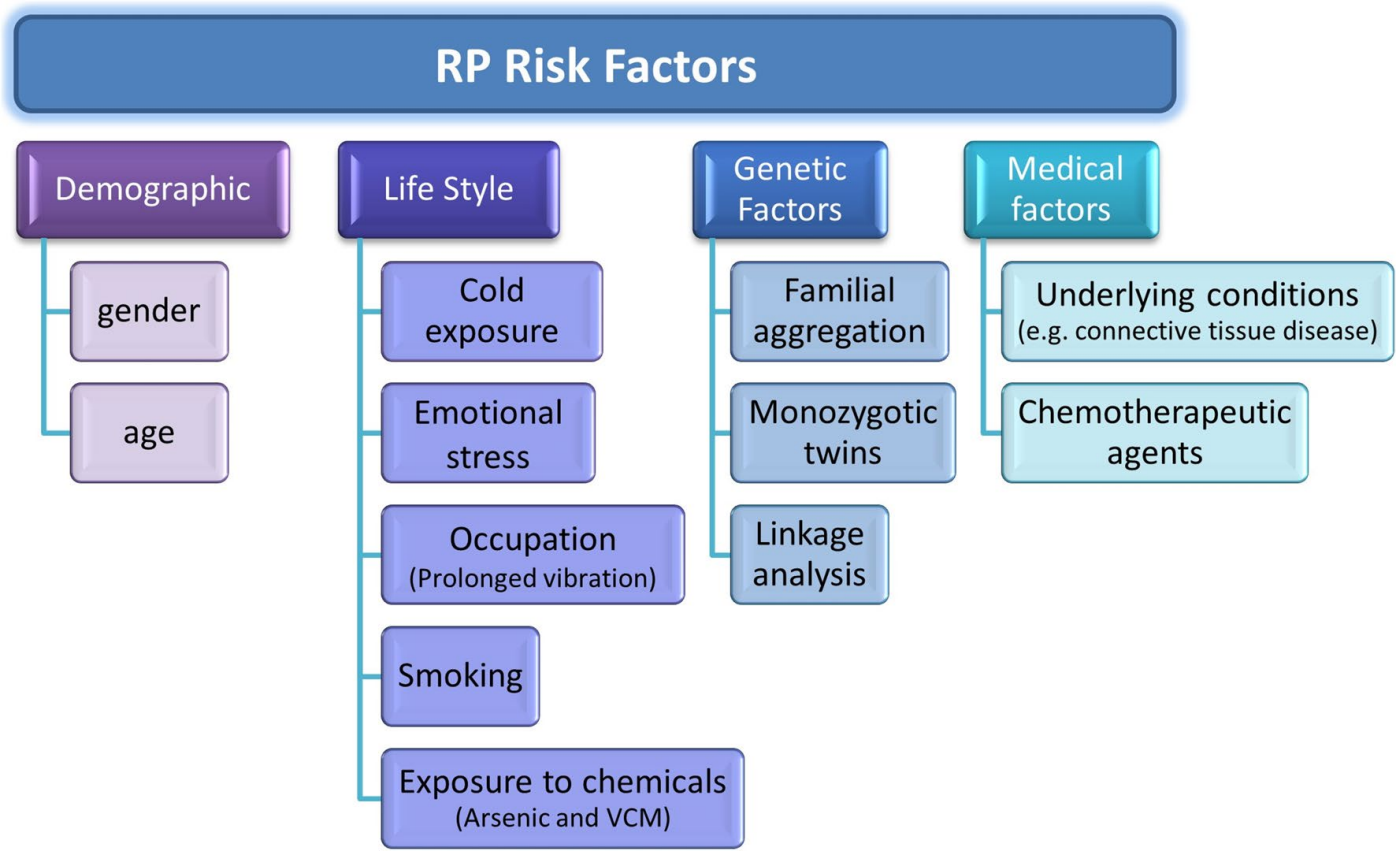
Risk factors of Raynaud’s phenomenon. Demographic factors, intrinsic to the patient, encompass gender and age. Environmental factors, inseparably linked to one's modus vivendi, comprise various stimuli that may precipitate or aggravate Raynaud’s phenomenon. These include cold temperatures, psychologic stressors, vibratory and mechanical insults, and chemical agents such as nicotine and vinyl chloride monomer. Numerous studies also support the involvement of a genetic factor in Raynaud’s phenomenon incidence. Medical factors, whether stemming from underlying pathologies or iatrogenic in nature, can modulate both the risk and severity of Raynaud’s phenomenon. Certain pharmacologic agents, particularly chemotherapeutics like gemcitabine, have also been implicated in the pathogenesis of Raynaud’s phenomenon

Raynaud’s phenomenon is influenced by several risk factors that predispose individuals to this vascular disease or exacerbate its symptoms (Fig. 2). These factors can be categorized into four groups: demographic, environmental, genetic, and medical.

### Demographic factors

The preponderance of Raynaud’s phenomenon in females is incontrovertible, with incidence markedly elevated in females vis-à-vis their age-matched males (Fardoun et al. 2016, 2024). This gender disparity is intimately linked to estrogen, whose role in Raynaud’s phenomenon pathogenesis has been illustrated. Age, too, plays a pivotal role: primary Raynaud’s phenomenon typically manifests in reproductive age females (15 and 30 years) while secondary Raynaud’s phenomenon is diagnosed later as it is often associated with an underlying pathologic conditions or lifestyle factors (Temprano 2016; Belch et al. 2017; Prete et al. 2014). Other minor factors include low body mass index which increases the susceptibility to cold (Nawaz et al. 2022), and frostbite sequelae, which damage tissues and elevate Raynaud’s phenomenon risk (Belch et al. 2017).

### Environmental and lifestyle factors

Cold exposure is a major catalyst and trigger of Raynaud’s phenomenon, as it evokes spatial translocation of α_2C_-AR to the membrane of arteriolar SMCs. This mobilization engenders contractile responses, and subsequently culminates in vasoconstriction (Chotani et al. 2000). As such, living in colder climates augments the propensity for vasospastic attacks and Raynaud’s phenomenon manifestation (Plissonneau Duquene et al. 2015). This explains the markedly higher incidence of Raynaud’s phenomenon in regions of colder climate (Plissonneau Duquene et al. 2015).

Emotional stress is another potent trigger as it activates the sympathetic system to engender vasoconstriction and potentially Raynaud’s phenomenon (Brown et al. 2001). These psychogenic and thermal stressors act as sympathetic stressors, as they induce noradrenaline-mediated vasoconstriction (Fardoun et al. 2016). Conversely, localized stressors, such as prolonged and recurrent exposure to mechanical insults from vibrating tools, can potentiate digital vasospasm, thereby augmenting the risk of secondary Raynaud’s phenomenon (White et al. 2004). A salient example is the vibration-induced white finger, also known as hand-arm vibration syndrome (White et al. 2004). Thus, individuals in construction and manufacturing are especially vulnerable to vibration-induced vascular sequelae. This occupational hazard underscores Raynaud’s phenomenon’s multifaceted etiology and highlights the interplay between environmental factors and vascular dysregulation.

Chemicals stressors are also culprits in instigating Raynaud’s phenomenon. For example, tobacco smoking heightens the proclivity for Raynaud’s phenomenon due to its deleterious impact on vascular integrity (Garner et al. 2015). Indeed, nicotine, a principal alkaloid in tobacco, is a potent vasoconstrictor that can trigger or exacerbate Raynaud’s phenomenon vasospastic attacks (Cardelli and Kleinsmith 1989; Cherniack et al. 2000; Jackson 2006). Another chemical stressor is vinyl chloride monomer (VCM), used in poly vinyl chloride plastic. A study involving 761 retired workers exposed to VCM demonstrated a significant association between VCM and Raynaud’s phenomenon manifestation (Lopez et al. 2013; Fontana et al. 2006). While VCM’s deleterious impact on digital microcirculation is well-established (Falappa et al. 1982), this study is particularly striking as it underscores the residual, long-term sequelae of VCM exposure. This emphasizes the persistent nature of chemically-induced vascular perturbations in Raynaud’s phenomenon etiology, underscoring the necessity for sustained vigilance in occupational health surveillance.

Another chemical compound in Raynaud’s phenomenon onset is arsenic (Fardoun et al. 2016; Lagerkvist et al. 1986). A positive correlation has been observed between arsenic exposure and the prevalence of peripheral vascular symptoms, including Raynaud’s phenomenon, in regions with arsenic-contaminated drinking water (Lagerkvist et al. 1986). Notably, arsenic-exposed smelter workers exhibit heightened vasospastic reactivity in the fingers, a hallmark of Raynaud’s phenomenon pathophysiology (Lagerkvist et al. 1986; Mereto et al. 1975). This underscores the potential for chronic arsenic exposure to engender functional alterations in the microvasculature, further linking environmental toxins to Raynaud’s phenomenon development.

### Genetic factors

Familial predisposition to Raynaud’s phenomenon has also been demonstrated in studies showing a higher incidence in families or probands compared to controls (Tan and Arnett 2000; Freedman and Mayes 1996). This familial aggregation, coupled with concordance in monozygotic twins, suggests a genetic component in Raynaud’s phenomenon etiology (Freedman and Mayes 1996; Oskay and Olmez 2004; Pistorius et al. 2006). However, definitive genetic confirmation remains elusive. Initial investigations into candidate genes, including the β-subunit of the muscle acetylcholine receptor and serotonin receptors 1B and 1E, failed to identify causative mutations but revealed susceptibility loci potentially linked to Raynaud’s phenomenon (Susol et al. 2000).

Subsequent research identified other genetic factors. For instance, polymorphisms in glutathione S-transferase M1 and T1 genes synergistically increased Raynaud’s phenomenon risk in VCM-exposed individuals (Fontana et al. 2006). Moreover, variations in the NOS1 gene, encoding nitric oxide synthase involved in vasodilation after cold exposure, have also been linked to Raynaud’s phenomenon (Munir et al. 2018). A recent genome-wide association study conducted on 5147 Raynaud’s phenomenon patients identified three novel genomic regions linked to increased Raynaud’s phenomenon risk (Hartmann et al. 2023). However, limitations in the study’s cell and vessel models prevent conclusive findings.

### Clinical factors

Secondary Raynaud’s phenomenon often arises as a sequela of underlying conditions, particularly connective tissue disorders (CTDs) such as systemic sclerosis (SSc), systemic lupus erythematosus, and Sjogren’s syndrome (Belch et al. 2017). Hematologic conditions like cryoglobulinemia, paraproteinemia, and cryofibrinogenemia also predispose to Raynaud’s phenomenon by increasing viscosity and reducing digital perfusion (Hughes et al. 2015, 2020). Structural vascular abnormalities, both obstructive (e.g., brachiocephalic trunk disease, atherosclerosis) (Temprano 2016) or compressive (e.g., thoracic outlet syndrome) (Hughes et al. 2020) contribute to Raynaud’s phenomenon etiology. Comorbidities affecting hand vasculature such as carpal tunnel syndrome and hand arm vibration syndrome further elevate Raynaud’s phenomenon risk (Cooke et al. 2022). In addition, thyroid disorders were also found to increase risk to Raynaud’s phenomenon (Shagan and Friedman 1976, 1980; Belch et al. 2017).

Pharmacologic agents, especially chemotherapeutics, can evoke or exacerbate Raynaud’s phenomenon through diverse mechanisms, including endothelial injury, neurotoxicity, and thrombotic microangiopathy (Venat-Bouvet et al. 2003; Holstein et al. 2010). For instance, gemcitabine has been associated with finger swelling whitening, ischemia and necrosis (Carmichael 1998; Clowse and Wigley 2003; D’Alessandro et al. 2003). Other antineoplastic drugs, including bleomycin, vinblastine, carboplatin, doxorubicin, and vincristine, are also linked to increased Raynaud’s phenomenon risk (Saif et al. 2016; Gottschling et al. 2004; Hansen and Olsen 1989). The putative mechanisms underlying this predisposition include neurotoxicity and sympathetic hyperreactivity (Chant 1987; Olsen et al. 1987).

Various non-chemotherapeutic medications also potentiate Raynaud’s phenomenon risk. β-adrenoreceptor blockers, clonidine, and dopaminergic agonists are implicated due to their vasoconstrictive properties (Khouri et al. 2016; Laboe et al. 2021). Interferon therapy, which augments blood viscosity and promotes vasoconstriction, is also associated with Raynaud’s phenomenon (Linden 1998). Tyrosine kinase inhibitors have also been implicated, although the underlying mechanism remains elusive (Khouri et al. 2016). Taken together, this multifactorial etiology of secondary Raynaud’s phenomenon underscores its complexity pathophysiology, warranting thorough clinical evaluation and pharmacovigilance in affected individuals.

## Treatment of Raynaud’s phenomenon

The multifaceted etiopathology of Raynaud’s phenomenon presents significant challenges for developing efficacious treatments (Wigley 2002). The lack of a reliable animal model further complicates this effort. Hence, no definitive Raynaud’s phenomenon treatment has been yet approved by the Food and Drug Administration (FDA). However, current strategies focus on alleviating symptoms, reducing the frequency and severity of vasospastic episodes, and preventing tissue damage.

**Fig. 3:**
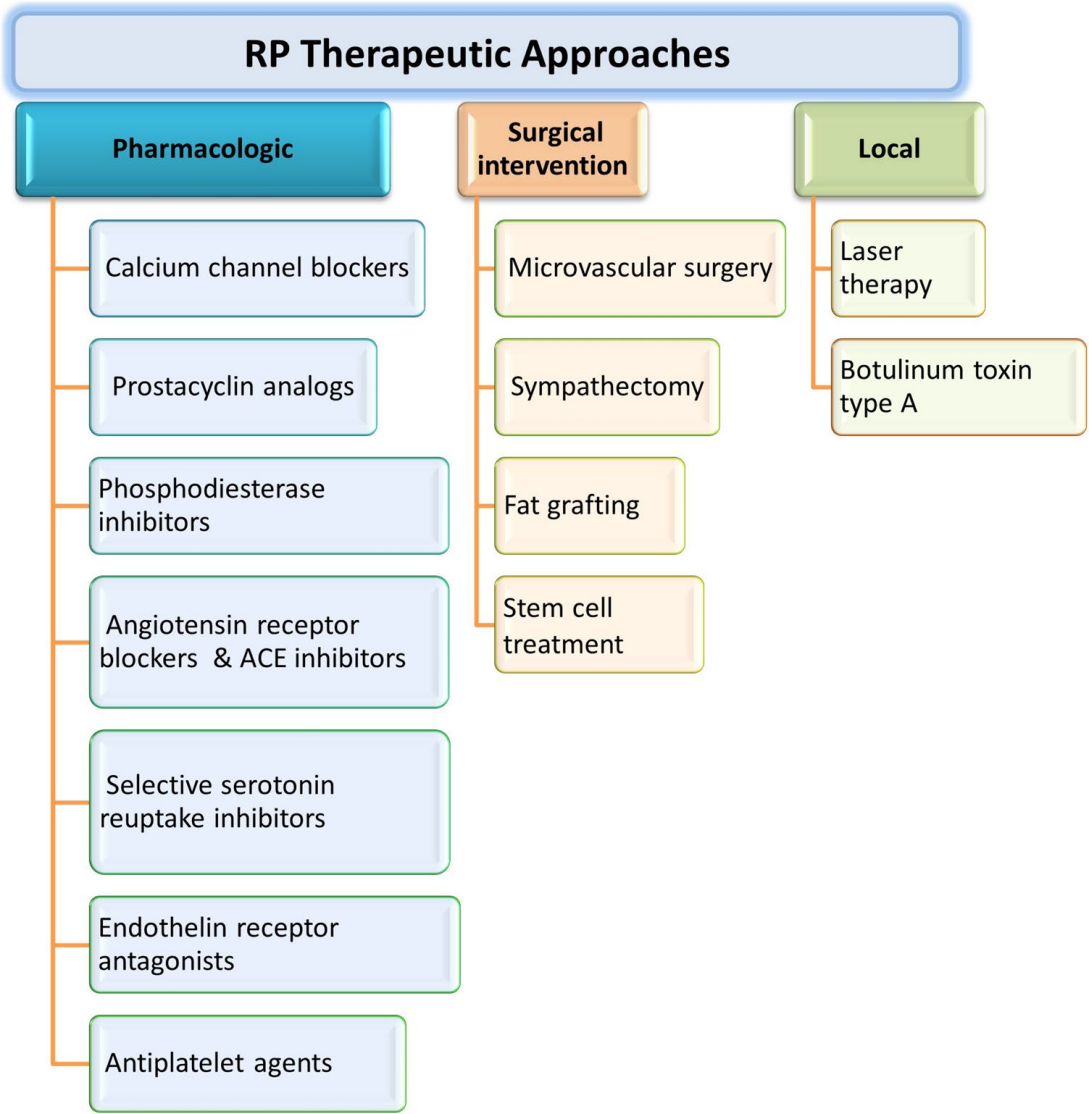
Raynaud’s phenomenon therapeutic approaches. Several approaches are employed to treat RP. In the pharmacologic approach, drugs such as calcium channel blockers or prostacyclin analogs are prescribed. Different drugs have different efficiencies that may vary depending on the patient. More sever Raynaud’s phenomenon cases require surgical intervention such as sympathectomy or fat grafting. Unconventional approaches including laser therapy and botulinum toxin injection are also effective in Raynaud’s phenomenon treatment

The therapeutic armamentarium for Raynaud’s phenomenon encompasses a spectrum of approaches, as summarized in Fig. 3. The first-line pharmacologic intervention for Raynaud’s phenomenon is calcium channel blockers (CCBs) such as nifedipine and amlodipine, which suppress vasoconstriction and somewhat promote vasodilation, alleviating symptoms (Thompson and Pope 2005). Oral nifedipine is particularly the usual recommendation (Del Galdo et al. 2025). Nifedipine is typically administered at oral doses of 30–60 mg/day, with an elimination half-life of 2–4 h and a peak plasma concentration occurring within 1–2 h post-administration, reflecting its rapid absorption in immediate-release formulations (Khan et al. 2025). In contrast, amlodipine is prescribed at lower doses (5–10 mg/day) but exhibits a significantly prolonged half-life of 30–50 h, enabling once-daily dosing and stable plasma concentrations over time (Bulsara et al. 2025). Both drugs undergo hepatic metabolism via cytochrome P450 enzymes, primarily CYP3A4, but differ in excretion pathways. Nifedipine is eliminated through both renal and fecal routes, with only trace amounts of the unchanged drug detectable in urine. Amlodipine, however, is primarily excreted in urine as inactive metabolites, with approximately 10% of the unchanged drug renally cleared. Recently, a novel CCB has shown efficacy in treating Raynaud’s phenomenon secondary to scleroderma even in non-responders to other CCBs (Bixio et al. 2024). Evidence suggests that CCBs reduce the severity and frequency of vasospastic attacks, though findings on duration are mixed (Ennis et al. 2016; Rirash et al. 2017).

Prostacyclin analogs, especially for patients with acute ischemic injury to the digits and ulcers, are also recommended (Scorza et al. 2001; Milio et al. 2006; Kawald et al. 2008; Cruz et al. 2016). Being potent vasodilators and platelet anti-aggregants, prostacyclin analogs alleviate the severity and frequency of Raynaud’s phenomenon vasospastic attacks. Commonly used drugs of this class include iloprost, epoprostenol, and treprostinil.

Iloprost and epoprostenol are both administered intravenously, with typical dosing ranges of 0.5–2 ng/kg/min and 2–25 ng/kg/min, respectively. Iloprost exhibits a half-life of 20–30 min, while epoprostenol’s rapid metabolism results in a notably shorter half-life of 3–5 min (Zhang et al. 2016). Iloprost undergoes hepatic metabolism, with elimination primarily via renal and pulmonary pathways, whereas epoprostenol is predominantly metabolized by the lungs. Treprostinil offers flexible administration routes, including subcutaneous and intravenous delivery, at doses of 1–2 ng/kg/min. Its extended half-life of 4–6 h and hepatic metabolism distinguish it from shorter-acting analogs.

The most recent recommendations call for the use of intravenous iloprost as a second-line therapy if oral therapy fails (Del Galdo et al. 2025). Recently, selexipag, a prostanoid receptor agonist, was shown to ameliorate Raynaud’s phenomenon -induced severe digital ischemia (Langleben et al. 2021), and the ameliorative effect of selexipag on SSc digital vasculopathy was sustained for a year (Di Battista et al. 2024). However, a multicenter, double-blind, randomized, placebo-controlled trial reported that selexipag did not reduce the number of attacks (Denton et al. 2017).

Phosphodiesterase inhibitors like vardenafil and sildenafil appear to improve digital blood flow and reduce Raynaud’s phenomenon attack severity (Lee et al. 2014; Caglayan et al. 2006). These drugs outperform amlodipine, a CCB, in enhancing digital blood flow (Lee et al. 2014). Vardenafil and sildenafil are both orally administered phosphodiesterase-5 inhibitors used to treat erectile dysfunction. Vardenafil is typically dosed at 5–20 mg, while sildenafil is administered at 25–100 mg. Vardenafil exhibits a half-life of 4–5 h, whereas sildenafil’s elimination half-life ranges between 3–5 h (Kim et al. 2025). Both drugs undergo hepatic metabolism, primarily mediated by the CYP3A4 enzyme, and are excreted through fecal and urinary pathways. While their pharmacokinetic profiles share similarities in clearance mechanisms, vardenafil’s slightly longer half-life and higher biochemical potency (requiring lower doses) distinguish it from sildenafil. The most recent guidelines retained the previous recommendation for the use of phosphodiesterase 5 inhibitors for the treatment of digital ulcers (Del Galdo et al. 2025).

Most international guidelines, including those from the European League Against Rheumatism (EULAR), the American College of Rheumatology (ACR), and the British Society for Rheumatology (BSR), converge on a unified, stepwise approach to managing Raynaud’s phenomenon (Belch et al. 2017). At the forefront of pharmacologic treatment are CCBs, particularly dihydropyridines such as nifedipine, which are widely recommended as the first-line therapy. Their vasodilatory effects have been shown to reduce the frequency of vasospastic attacks and alleviate symptom severity effectively. For patients who do not respond adequately to CCBs or who cannot tolerate them, phosphodiesterase type 5 (PDE-5) inhibitors like sildenafil are commonly advised as second-line options, providing additional symptomatic relief. While the sequence of treatments beyond these core agents may vary across different guidelines, the consistent emphasis on CCBs and PDE-5 inhibitors highlights their central role in managing Raynaud’s phenomenon. Importantly, these pharmacological interventions are always combined with general conservative measures, including avoiding cold exposure and smoking cessation, which are critical in minimizing vasospastic triggers and improving overall outcomes. This integrated approach reflects a broad consensus among leading rheumatology societies, underscoring the importance of both medication and lifestyle modifications in effective disease management (Belch et al. 2017, van den Hoogen et al. 2013).

Angiotensin receptor blockers (ARBs) and angiotensin converting enzyme (ACE) inhibitors may be used as Raynaud’s phenomenon treatment due to their ability to reduce vascular tone, thus reducing in the severity and frequency of spastic episodes (Dziadzio et al. 1999; Maddison 2002). For instance, patients with primary but not secondary Raynaud’s phenomenon taking losartan, an ARB, showed a significant reduction in the frequency and intensity of attacks (Maddison 2002). However, another study reported no improvement in Raynaud’s phenomenon patients taking quinapril, an ACE inhibitor, for 3 years (Gliddon et al. 2007). As such, ARBs are recommended more than ACE inhibitors for the treatment of Raynaud’s phenomenon (Linnemann and Erbe 2016). Losartan, an ARB, is administered orally at doses ranging from 25–100 mg daily. It has a half-life of approximately 2 h and undergoes hepatic metabolism primarily via cytochrome P450 enzymes (CYP2C9 and CYP3A4). The drug is excreted through both renal and fecal routes, with its active metabolite (E 3174) contributing significantly to its therapeutic effects and exhibiting a prolonged half-life of 6–9 h. Quinapril, an ACE inhibitor, is typically prescribed at doses of 10–40 mg daily and has a half-life of around 2 h. It is metabolized in the liver to its active form, quinaprilat, which is primarily eliminated via renal excretion. While both agents share similarities in hepatic metabolism and short parent compound half-lives, their clearance pathways differ, with losartan’s dual excretion contrasting quinapril’s predominant renal elimination.

Selective serotonin reuptake inhibitors (SSRIs) have shown mixed efficacy (Khouri et al. 2017). A study showed that fluoxetine, an SSRI, lead to significant improvement in the thermographic response to cold in female Raynaud’s phenomenon patients (Coleiro et al. 2001). However, a more recent study concluded that there is still a discrepancy in literature regarding the use of SSRI in Raynaud’s phenomenon treatment and that more evidence is needed to come to a final conclusion (Khouri et al. 2017). Fluoxetine is orally administered at a typical dose of 20–60 mg/day. Its half-life is 4–6 days. Fluoxetine is metabolized in the liver and excreted primarily in the urine.

Endothelin receptor antagonists (ETRAs) are used in cases of Raynaud’s phenomenon secondary to SSc. Patients usually suffer from microvascular damage and ulcers, which lead to increased release of the vasoconstrictor endothelin-1. As such, inhibiting endothelin using ETRAs treated SSc and decreased the severity and frequency of vasospastic attacks in these patients (Poredos and Poredos 2016). The first case study on the therapeutic effect of ETRA on Raynaud’s phenomenon reported decreased vasospastic attacks following bosentan administration (Selenko-Gebauer et al. 2006). This finding was supported in later studies reporting the same therapeutic effect (Funauchi et al. 2009; Nguyen et al. 2010; Arefiev et al. 2011). Bosentan, a known ETRA, is orally administered at a dose of 62.5–125 mg twice daily. Its half-life is 5 h. Bosentan is metabolized in the liver and eliminated in the feces.

The aforementioned pharmacologic treatment has been reported to cause certain side effects, such as headache and dizziness, prompting many patients to seek alternative therapies in an effort to circumvent such complications. Among these alternatives, herbal medicine has emerged as a popular approach for managing Raynaud’s phenomenon. Specifically, Ginkgo biloba extract was initially found to improve the condition by enhancing blood circulation and reducing vasospasm (Muir et al. 2002). However, this finding was challenged a decade later by a randomized trial which, while affirming the safety of Ginkgo biloba use in patients with Raynaud’s phenomenon, did not demonstrate a statistically significant therapeutic benefit (Bredie and Jong 2012). Other herbal treatments have also been explored. Jiejing Tongmi Tang has been reported to alleviate symptoms of Raynaud’s phenomenon (Zhang et al. 2020). In addition, a combination of two Chinese herbal formulas, Buyang Huanwu Tang and Danggui Sini Tang, showed therapeutic effects that surpassed those of nifedipine (Zhang et al. 2020). In contrast, another combination involving Duhuo-Tisheng Tang and Danggui-Sini did not improve the digital vascular response in patients with Raynaud’s phenomenon (Wu et al. 2008). Interestingly, some studies suggest that omega-3 fatty acids found in fish oil may provide beneficial effects by reducing the severity of symptoms (DiGiacomo et al. 1989). While these findings offer promising insights into the potential therapeutic effects of herbal and natural remedies for Raynaud’s phenomenon, more rigorous and well-designed studies are necessary to confirm their efficacy, determine appropriate dosages, and establish standardized treatment protocols (Muir et al. 2002).

Accumulating evidence supports the therapeutic effect of botulinum toxin type A (BTX-A) on Raynaud’s phenomenon patients (Sycha et al. 2004; Van Beek et al. 2007; Fregene et al. 2009). For instance, locally injecting BTX-A to Raynaud’s phenomenon patients significantly reduced the frequency of vasospastic attacks, alleviated pain sensation, and decreased ulcers and necrosis (Medina et al. 2018). In addition, Doppler study showed improved blood flow 30 min after infiltration of BTX-As (Neumeister et al. 2009). Most recently, the largest real-life patient cohort on botulinum toxin for Raynaud’s phenomenon provide evidence supporting the use of BTX (Pinto-Pulido et al. 2025). The mechanism of action of BTX-A in the context of Raynaud’s phenomenon is not fully understood. BTX-A is known to cause vasodilation by paralyzing the acetylcholine-mediated arterial muscles (Stone et al. 2012); however, this mechanism does not explain the healing experienced by Raynaud’s phenomenon patients. BTX-A also reduces vasoconstriction and pain sensation by inhibiting norepinephrine release and alpha-adrenergic receptor expression (Setler 2002), as well as by suppressing the release of substance P (Kim et al. 2015). BTX-A is administered via local injection at varying doses, ranging from 50 to 100 units per site, depending on the severity of the symptoms (Dhaliwal et al. 2018; Medina et al. 2018). The duration of action is typically 3–6 months. BTX-A is excreted through the urinary system in the form of Botox metabolites. It is important to mention that one of the challenges of using these injections is that they need to be repeated periodically. Moreover, some patients may develop resistance to BTX, and hence, render the injections relatively ineffective.

Raynaud’s phenomenon patients with severe symptoms and critical ischemia that may precipitate tissue necrosis resort to surgical intervention. In cases where pharmacologic therapy showed no or limited efficacy in alleviating Raynaud’s phenomenon symptoms; surgical intervention, albeit invasive, was successful in reducing pain and healing ulcers (Landry 2013). This includes sympathectomy and microvascular surgery. While sympathectomy is performed to disrupt sympathetic nerve signals thereby reducing vasospasm (Kaada 1982), microvascular reconstructive surgery is performed in severe cases to restore blood flow and preserve tissue viability.

Fat grafting is a new and unconventional surgical intervention for Raynaud’s phenomenon. In a yet unknown mechanism, fat grafting decreased cold attacks and pain sensation in addition to improving ulcers (Bank et al. 2014). Neoangiogenesis and stem cells are thought to be involved in the alleviative effects observed following fat grafting (Bank et al. 2014). Recently, revolutionary stem cell treatment has been employed to treat Raynaud’s phenomenon patients. Hematopoietic stem cells harvested from patient’s bone marrow are then injected into arteries that nourish fingers and toes. This stem cell treatment enhanced blood flow and alleviated pain sensation in Raynaud’s phenomenon patients. In addition, it is a personalized treatment that evades rejection or side effects, and more importantly, it is less invasive than the aforementioned surgical interventions.

There is an increasing interest in laser-based therapy for Raynaud’s phenomenon. Several studies showed that laser therapy reduced the severity and frequency of vasospastic attacks in Raynaud’s phenomenon patients. Multiwave Locked System (MLS) laser therapy appear to decrease the number, duration, and intensity of Raynaud’s phenomenon attacks, and it can reduce the avascular areas in affected organs (Kuryliszyn-Moskal et al. 2015). The mechanism of action of MLS remains unclear (al-Awami et al. 2001); however, it is speculated that MLS, by interacting with deeply located tissues, may affect cellular membrane permeability, vessel walls, and peripheral nervous system (Kuryliszyn-Moskal et al. 2015). This leads to anti-inflammatory, anti-edematous effect at the level of blood vessels as well as analgesic effect.

**Table:** 

Therapy	Examples	Mechanism of action	Efficacy
Calcium channel blockers (CCBs)	Nifedipine, amlodipine, novel CCB (Bixio et al. 2024)	Vasodilation via calcium channel inhibition	Reduces frequency/severity; mixed data on duration
Prostacyclin analogs	Iloprost, epoprostenol, treprostinil, selexipag	Potent vasodilation, platelet anti-aggregation	Recommended for ischemic injury/ulcers; selexipag has mixed evidence
Phosphodiesterase 5 inhibitors	Sildenafil, vardenafil	Increased cGMP leading to vasodilation	Improves digital perfusion, reduces RP attacks
Angiotensin receptor blockers (ARBs)	Losartan	Reduces vascular tone via angiotensin receptor blockade	Reduces RP symptoms in primary RP; not effective in SSc
ACE inhibitors	Quinapril	Decreases angiotensin II production	No benefit seen in long-term study
SSRIs	Fluoxetine	Inhibits serotonin reuptake, affects vasoconstriction	Mixed results urging more dedicated studies
Endothelin receptor rntagonists (ETRAs)	Bosentan	Blocks endothelin-1 vasoconstriction	Effective in SSc with ulcers; supported by multiple studies
Botulinum toxin type A (BTX-A)	BTX-A (Botox)	Reduces vasoconstriction and pain via neurotransmitter blockade	Reduces attacks, pain, and ischemia; supported by imaging and cohort data
Sympathectomy	Thoracic sympathectomy	Interrupts sympathetic vasoconstrictive signaling	Effective when medical therapy fails; invasive
Microvascular surgery	Digital artery bypass	Restores blood flow in ischemic tissues	Used in severe ischemia; invasive but effective
Fat grafting	Autologous fat transfer	Neoangiogenesis, possible stem cell activity	Improves pain and ulcers; mechanism unclear
Stem cell therapy	Autologous hematopoietic stem cells	Promotes angiogenesis, improves circulation	Improves blood flow and pain; personalized and less invasive
Laser therapy (MLS)	Multiwave Locked System (MLS) laser	Unknown; possibly membrane effects and neurovascular modulation	Reduces attack number and severity; mechanism unclear

## Conclusion and perspectives

The mosaic etiology of Raynaud’s phenomenon has rendered the discovery of a definitive treatment rather difficult. Prophylaxis remains the cornerstone of Raynaud’s phenomenon management, particularly in cases of primary Raynaud’s phenomenon. Lifestyle modifications to minimize Raynaud’s phenomenon triggers are paramount, including the avoidance of cold temperatures, psychologic stressors, and tobacco use. Patients are also advised to engage in regular physical activity to improve circulation. Without a definitive treatment, the optimal approach to alleviate Raynaud’s phenomenon symptomatology combines these preventive measures with pharmacologic interventions.

α_2C_-AR, the major mediator of local cold-induced vasoconstriction, is expressed in many regions of the brain and implicated in presynaptic regulation of the heart. Thus, targeting α_2C_-AR to treat Raynaud’s phenomenon is fraught with potential risks. Surprisingly, a recent study reported that a selective α_2C_-AR antagonist used to treat diabetic foot ulcers was efficient and safe (Kapsa et al. 2022). Notably, this antagonist has a limited ability to cross the blood–brain barrier. Further investigation into the efficacy of such antagonists in inhibiting α_2C_-AR-mediated vasoconstriction, and consequently treating Raynaud’s phenomenon, would not only be tempting but also of significant scientific value.

Focusing on molecular targets predominantly expressed in arteriolar SMCs may offer the most promising path for novel Raynaud’s phenomenon therapies. As such, investigating the mechanisms governing α_2C_-AR expression and trafficking may reveal potential molecular targets. Another attractive strategy is drug repurposing; for instance, cilostazol, originally used for intermittent claudication, is being explored as a potential Raynaud’s phenomenon treatment (El-Hachem et al. 2021). While drug repurposing shows promise, it is not a radical therapy for Raynaud’s phenomenon and the current lines of treatment only alleviate its symptoms.

## Data Availability

Not applicable.
